# Geographic distance and pH drive bacterial distribution in alkaline lake sediments across Tibetan Plateau

**DOI:** 10.1111/j.1462-2920.2012.02799.x

**Published:** 2012-09

**Authors:** Jinbo Xiong, Yongqin Liu, Xiangui Lin, Huayong Zhang, Jun Zeng, Juzhi Hou, Yongping Yang, Tandong Yao, Rob Knight, Haiyan Chu

**Affiliations:** 1 State Key Laboratory of Soil and Sustainable Agriculture, Institute of Soil Science, Chinese Academy of SciencesNanjing 210008, China; 2 Key Laboratory of Tibetan Environment Changes and Land Surface Processes, Institute of Tibetan Plateau Research, Chinese Academy of SciencesBeijing 100085, China; 3 Department of Chemistry and BiochemistryBoulder, CO 80309, USA.; 4 Howard Hughes Medical Institute, University of ColoradoBoulder, CO 80309, USA.

## Abstract

Continent-scale biogeography has been extensively studied in soils and marine systems, but little is known about biogeographical patterns in non-marine sediments. We used barcode pyrosequencing to quantify the effects of local geochemical properties and geographic distance for bacterial community structure and membership, using sediment samples from 15 lakes on the Tibetan Plateau (4–1670 km apart). Bacterial communities were surprisingly diverse, and distinct from soil communities. Four of 26 phyla detected were dominant: *Proteobacteria*, *Bacteroidetes*, *Firmicutes* and *Actinobacteria*, albeit 20.2% of sequences were unclassified at the phylum level. As previously observed in acidic soil, pH was the dominant factor influencing alkaline sediment community structure, phylotype richness and phylogenetic diversity. In contrast, archaeal communities were less affected by pH. More geographically distant sites had more dissimilar communities (*r* = 0.443, *P* = 0.030). Variance partitioning analysis showed that geographic distance (historical contingencies) contributed more to bacterial community variation (12.2%) than any other factor, although the environmental factors explained more variance when combined (28.9%). Together, our results show that pH is the best predictor of bacterial community structure in alkaline sediments, and confirm that both geographic distance and chemical factors govern bacterial biogeography in lake sediments.

## Introduction

Understanding which environmental factors influence the variation in microbial communities across different scales is a key goal of ecology. Many studies show that contemporary environmental factors, such as trophic status (Hansel *et al*., 2008), soil pH (Rousk *et al*., 2010), metalloid contamination, and the rhizosphere (Xiong *et al*., 2010), influence microbial community structure. In contrast, and because of these large effects of local factors, links between microbial community structure and geographic distance remain elusive and controversial (Martiny *et al*., 2006), and are mainly linked to studies of single species, such as *Bacillus* (Connor *et al*., 2010), and *Cyanbacteria* (Bahl *et al*., 2011). Cho and Tiedje (2000) first showed that the genetic divergence of *Pesudomonas* isolates correlated with spatial distance at regional (5 m to 80 km) but not greater scales, whereas a larger-scale (1–1000 km) survey of microbial eukaryotes revealed a taxa–area relationship (Hillebrand *et al*., 2001). Similarly, microbial endemism (Whitaker *et al*., 2003), and distance effects on microbial spatial dispersal (Martiny *et al*., 2006), have been reported. However, there is still active debate about the relative contribution of geographic distance to microbial community dissimilarity in soils; it is unclear whether geographic distance is a trivial (Fierer and Jackson, 2006; Ramette and Tiedje, 2007), important (Zhou *et al*., 2008), or dominant factor (Ge *et al*., 2008) relative to local environmental factors.

Recent studies suggest a consensus that the microbial community structure and diversity correlate significantly with soil pH across local (Rousk *et al*., 2010), regional (Hollister *et al*., 2010) and continent (Fierer and Jackson, 2006; Lauber *et al*., 2009; Chu *et al*., 2010) scales. However, these studies focused on acidic soils, and did not directly test the relative contribution of pHand geographic distance to community structure. A meta-analysis showed that sediments are more phylogenetically diverse than any other environment type (Lozupone and Knight, 2007). However, limited evidence exists for spatial patterns in sediments, although dispersal would be expected to be lower than in soils (Martiny *et al*., 2006). Bacteria also use different strategies to survive acidic and alkaline conditions (Dilworth and Glenn, 1999; Yohannes *et al*., 2004). In the present work we, therefore, examine alkaline lake sediments, testing whether predictable relationships between bacterial communities and factors such as pH and geographic distance mirror those observed in soils.

The Tibetan Plateau is the Earth's largest (2 × 10^6^ km^2^) and highest (average ∼ 4500 m a.s.l.) plateau. It contains several thousand saline and alkaline lakes, and is remote from centres of human activity. The region has a dry climate, and salinity increases from south to north due to decreased annual precipitation (Yang *et al*., 2003). The Tibetan Plateau thus provides an excellent natural laboratory for studying microbial distribution patterns on a regional scale. Several studies have attempted to characterize microbial communities in individual hypersaline lake sediments based on techniques with limited resolution, phospholipid fatty acid and/or 16S rDNA clone libraries (Dong *et al*., 2006; Jiang *et al*., 2007). The higher throughput permitted by pyrosequencing allows us to better characterize the diversity in this understudied system and to test the direct effects of geographic distance and environmental factors. We collected pristine sediment samples from 15 alkaline lakes across the Tibetan Plateau, and used barcoded pyrosequencing to evaluate the bacterial communities with respect to three broad and related aims: (i) to explore the taxonomic diversity of the bacteria on Tibetan Plateau alkaline lake sediments, (ii) to determine key factors in shaping the bacterial communities distribution, environmental heterogeneity, geographic distances, or both? and (iii) to quantify their relative importance to bacterial community variation.

## Results

### Sediment geochemical characteristics

The major geographical and physicochemical characteristics of the lake sediments are summarized in Table S1. Sediment pH varied from 6.88 to 10.37. Pairwise distances between sampling sites ranged from 4 to 1670 km (Table S2). There was no autocorrelation (*P* = 0.862) observed between sediments pH and geographic distance. While C/N ratios were significantly correlated with altitude (*r* = 0.561, *P* = 0.019), and pH was positively (*r* = 0.506, *P* = 0.038) correlated with Mg^2+^.

### Distribution of taxa and phylotypes

**Fig. 1:**
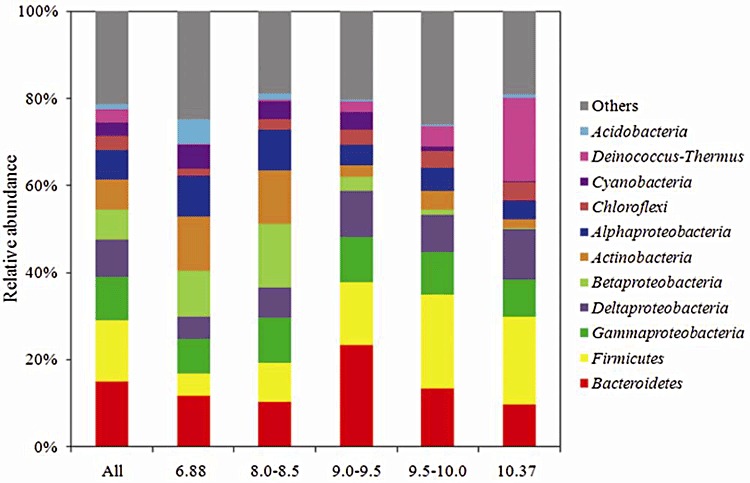
Relative abundances of the dominant bacterial phyla in all sediments combined, and in sediments separated according to pH categories. Relative abundances are based on the proportional frequencies of those DNA sequences that could be classified at the phylum level.

**Fig. 2:**
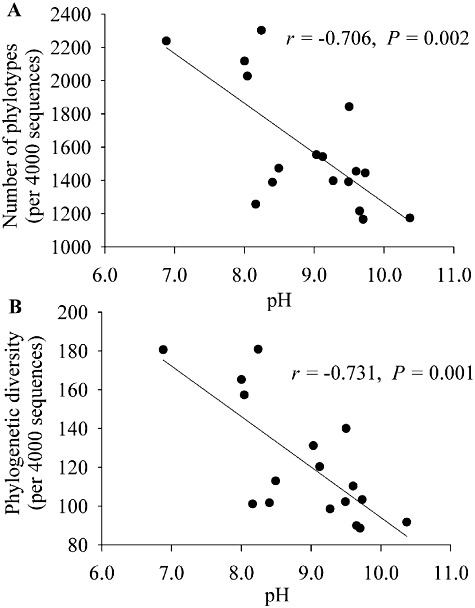
Sediment pH in relative to bacterial phylotype richness (A) and phylogenetic diversity (B) based on 97% sequence similarity in Tibetan Plateau lake sediments. The communities were randomly sampled at the 4000 sequences level.

Across all sediment samples, we obtained 139 714 quality sequences in total and 4173–19 050 sequences per sample (mean = 8218), and were able to classify 79.8% of those sequences. The dominant phyla across all sediments were *Bacteroidetes*, *Firmicutes*, *Gammaproteobacteria*, *Deltaproteobacteria*, *Betaproteobacteria*, *Actinobacteria* and *Alphaproteobacteria* (relative abundance > 5%), accounting for more than 61% of the bacterial sequences (Fig. 1). In addition, *Chloroflexi*, *Cyanobacteria*, *Euryarchaeota*, *Deinococcus-Thermus* and *Acidobacteria* were present in most sediments at low abundance, and 15 other rarer phyla were identified (Table S3). Interesting, 2.5% of the detected sequences were affiliated to *Halobacteria* within the *Archaea* (5.3% of classified OTUs). Alpha diversity, measured as the number of OTUs (≥ 97% similarity) detected per 4000 sequences was negatively correlated with sediment pH (*r* = −0.706, *P* = 0.002). Thus observed OTU richness doubled from high diversity (c. 2239 OTUs) at pH 6.88 and low diversity (c. 1174 OTUs) at pH 10.37 (Fig. 2A). This observation was confirmed using phylogenetic diversity, also negatively (*r* = −0.731, *P* = 0.001) correlated with pH (Fig. 2B), but not correlated with other sediment or site factors (*P* > 0.10 in all cases, Table S4), including salinity (Fig. S1). In contrast, the distribution of *Archaea* was not significantly correlated with pH (Fig. S2), indicating that bacterial community members are more affected by sediment pH than archaeal community members. This observation confirms patterns observed in a recent study, in which archaeal and bacterial communities responded differently to environmental gradients in hypersaline lake sediments (Swan *et al*., 2010).

### Bacterial community structure

**Fig. 3:**
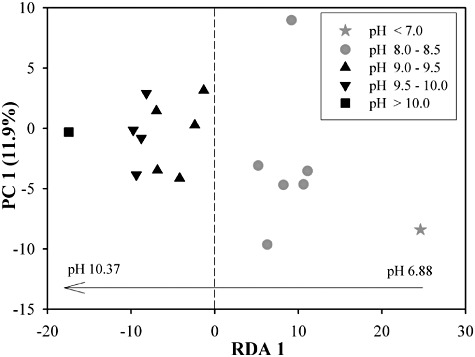
Principal component analyses (PCA) of the bacterial communities, with symbols coded by pH category.

**Fig. 4:**
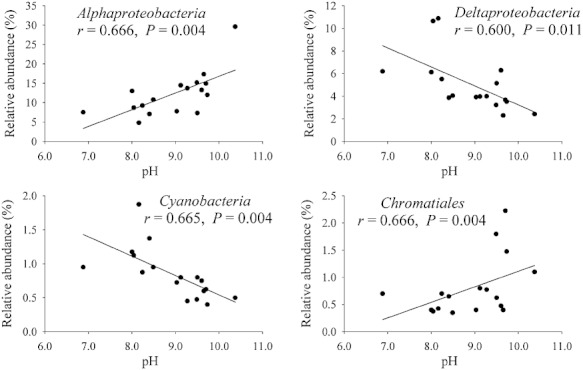
Relationships between relative abundances of dominant bacterial groups and sediment pH. Linear regressions were used to test Pearson correlation between each taxon's relative abundance and pH.

**Fig. 5:**
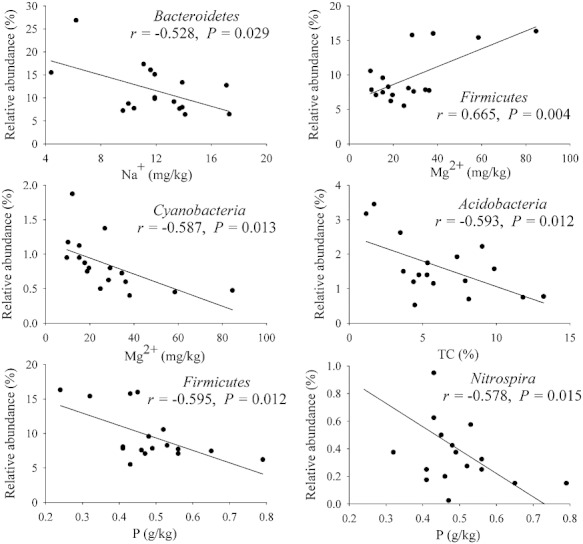
The relationships between relative abundances of dominant bacterial groups and sediment factors, including Na^+^, Mg^2+^, TC or TP respectively. Linear regressions were used to test Pearson correlation between the taxa's relative abundances and sediment factors.

Sediment pH strongly affected bacterial community structures across the gradient. The PCA (principal component analyses) biplot clearly shows that bacterial communities of different pH align along RDA1 axis (Fig. 3). This interpretation was supported by correlating bacterial community UniFrac distance with difference in sediment pH (*P* = 0.001, Mantel test). Other measured physical and geochemical factors, including salinity, altitude, C/N ratio of the samples, did not significantly improve the model over pH alone. The effect of sediment pH was evident even at a very coarse level of taxonomic resolution because the relative abundances of the dominant bacterial phyla (e.g. *Alphaproteobacteria* and *Deltaproteobacteria*), rarer phyla (e.g. *Cyanobacteria*), and classes (e.g. *Chromatiales*) were significantly correlated across the pH gradient (Fig. 4), although sometimes in opposite directions. For example, the relative abundance of *Deltaproteobacteria* decreased as pH increased, but *Alphaproteobacteria* increased. Some ions that correlate with pH, such as Na^+^ and Mg^2+^, showed significant correlations with the relative abundance of *Bacteroidetes*, *Firmicutes* or *Cyanobacteria*. Sediment TC (total carbon) and TP (total phosphorus) were significantly (*P* < 0.05 in all cases) correlated with the relative abundances of *Acidobacteria*, *Firmicutes* or *Nitrospira*, (Fig. 5). Taken together, the results strongly demonstrated that local sediment pH is, directly or indirectly, shaping the bacterial community structure among sites across the Tibetan Plateau lake sediments.

**Fig. 6:**
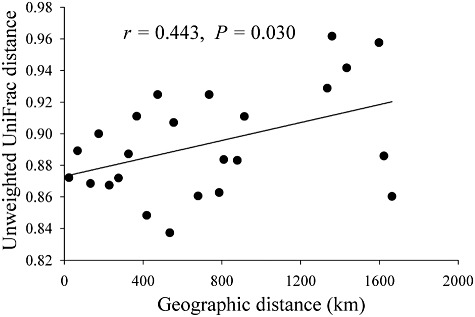
Correlation between community dissimilarities (unweighted UniFrac distance) and geographic distances. Means were calculated over discrete distance intervals of 50 km across all sampling sites, and data rescaled to facilitate plotting.

The samples contained 1166–2303 OTUs (phylotypes) per 4000 sequences (Fig. 2A); 49.0%–84.3% of phylotypes were unique to each sample, and few phylotypes overlapped in any pair of samples (0.24–12.27%, average 2.95%) (Table S5), indicating strict site endemism. In contrast, duplicate samples collected from the same lake shared more phylotypes: for example, KZC1 and KZC2 shared 11.5% of the OTUs, and those between LBC1 and LBC2 were 5.4% (Table S5). The unweighted UniFrac distances of bacterial communities (beta diversity) were significantly (*P* = 0.030) correlated with the geographic distance (Fig. 6). Partial Mantel tests further supported the distance effect when the effect of sediment environmental similarity matrix was held constant (*P* = 0.042). Together, the results revealed that spatial isolation affects community structure, presumably via dispersal, at this level of spatial and phylogenetic resolution.

### Linking bacterial communities to sediment properties and geographic distance

**Fig. 7:**
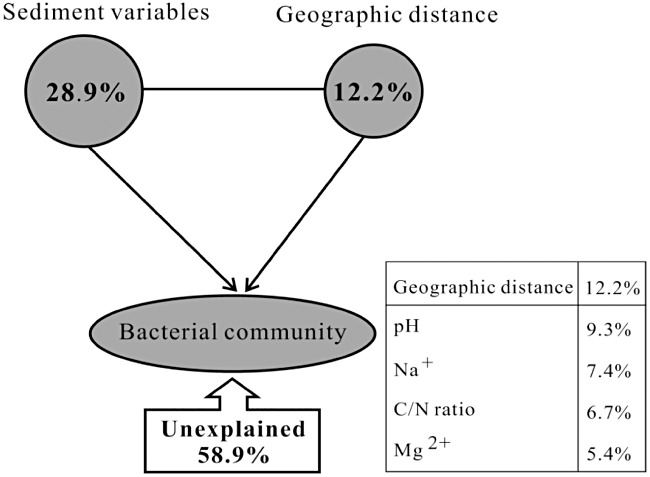
Variation partition analysis of the effects of geographic distance and sediment variables on the phylogenetic structure of bacterial communities.

Variance partitioning analysis (VPA) was performed to quantify the relative contributions of geographic distance and sediment environmental parameters to the taxonomic structure of the bacterial communities. A subset of environmental parameters (pH, Na^+^, Mg^2+^ and C/N ratio) was selected by the BioEnv procedure, which provides the highest Pearson correlation with bacterial communities. The combination of selected sediment properties and geographic distance showed a significant (*P* = 0.014) correlation with the bacterial community structures. These variables explained 41.1% of the observed variation, leaving 58.9% of the variation unexplained. The sediment properties explained 28.9% (*P* = 0.003), and geographic distance alone explained 12.2% (*P* = 0.029) variations, and no interaction effect was detected (Fig. 7). Although the sediment properties together explained more of the variation, geographic distance by itself explained 12.2% of the variation observed, more than any of the other 4 of the individual sediment variables (Fig. 7). Thus both sediment properties and geographic distance are important factors in shaping bacterial community structures.

## Discussion

Collecting sediment from pristine sites usually involved long drives over unfinished roads, making sampling hazardous and expensive. Consequently, most studies of microbial community composition in lake sediments have been limited to single lake (Jiang *et al*., 2007; Swan *et al*., 2010), preventing large-scale tests of bacterial spatial patterns in this habitat type.

In this study, we found that *Bacteroidetes* and *Firmicutes* were the main phyla in lake sediments, and high relative abundances of *Gammaproteobacteria* and *Deltaproteobacteria* were observed (Table S3); in contrast, these phyla are rare in soils (Chu *et al*., 2010; Griffiths *et al*., 2011), indicating that bacterial communities sediments are highly distinctive relative to soils (Lozupone and Knight, 2007).

The fundamental role of soil pH in shaping bacterial community structure has recently been demonstrated across North America (Lauber *et al*., 2009), in the Arctic (Chu *et al*., 2010), and across British soils (Griffiths *et al*., 2011). However, the samples examined in these studies were acidic to neutral, leaving open the question of whether pH also dominates the bacterial distribution in alkaline sediments, especially because some individual species can grow in environments spanning 4 pH units (Rosso *et al*., 1995). Here, we showed that the bacterial community structure, phylotype richness and phylogenetic diversity were primarily correlated with a single parameter, sediment pH (Figs 2 and 3, Table S4), although there was substantial variation in bacterial community structure across the range of surveyed sediments. The pH was the main factor controlling the bacterial community structure even within a limited range of 2.4 pH units if regardless the site pH 6.88 (Fig. 3), and nutrient concentrations were not significantly associated (Table S4). In addition, the ways in which specific phyla change across this pH gradient are similar to pH responses observed in soils, suggesting that they may be general across habitats. For instance, the relative abundance of *Alphaproteobacteria* has been shown to increase with higher pH (Chu *et al*., 2010; Rousk *et al*., 2010). The results imply that pH is a strong, universal predictor of bacterial community structure both for acidic soils and for alkaline lake sediments.

The distribution pattern of some phyla differed from previous observations. For example, previous work found *Acidobacteria* to be the dominant phylum across acidic soils (Lauber *et al*., 2009; Chu *et al*., 2010), but this phylum accounts for just 1.04% (average, Table S3) of the sequences in the current study. This result was expected, as many groups of *Acidobacteria* are more abundant at low pH (Griffiths *et al*., 2011) and the sediments in the present study are alkaline. Similar strong significant correlations between the relative abundance of *Acidobacteria* and *Bacteriodetes* and pH have been reported in soils from different biomes (Lauber *et al*., 2009; Chu *et al*., 2010), but were not detected in the Tibetan Plateau lake sediments. It should be noted that importance of pH in structuring bacterial communities does not completely erase the influence of other sediment or local features in driving the distribution of particular bacterial phyla. Indeed, we observed that TC and TP, Na^+^ and Mg^2+^, were significantly correlated with some dominant or subdominant phyla, matching the results obtained in earlier studies (Hollister *et al*., 2010; Griffiths *et al*., 2011). In particular, the relative abundances of *Bacteroidetes* and *Acidobacteria* were negatively correlated with Na^+^ and TC respectively. Na^+^ ions are critical to alkaliphilic bacteria (e.g. *Bacteroidetes*) as proton replacements to cope with high external pH (Skulachev *et al*., 1999). These phyla also play important roles in soil, where their relative abundances correlate with carbon mineralization rate (Fierer *et al*., 2007). Therefore, the changes in these dominant taxa over the pH gradient likely have consequences for ecosystem functions.

Salinity displays a strong gradient along our sampling sites. Given the key role of salinity in shaping bacterial community both in lake water (Wu *et al*., 2009) and globally (Lozupone and Knight, 2007), we expected to see a strong influence of salinity on the microbial communities in these sediments. However, there was no significant correlation between lake water salinity and bacterial diversity (Fig. S1, Table S4). Although the salinity of surface sediment was not directly measured, it is typically correlated with the lake water salinity because of its near water saturation. Similarly, Jiang and colleagues (2007) showed that the succession of proteobacterial groups along the salinity gradient was typically observed in free-living bacterial communities but not in sediments, and a recent study performed in hypersaline soils and sediments showed that site pH and organic carbon rather than salinity were the primary factors in controlling the bacterial structure on this local scale (Hollister *et al*., 2010). Thus, the main factors driving bacterial community structure in sediments differ in the details from those in lake water and soil. Our results emphasize the requirement of comparing the bacterial distribution pattern among local soils, lake water and sediments simultaneously in future work.

An issue relevant to our study, and to many other biogeographic studies, is whether spatial distance correlates strongly with genetic variation (Ramette and Tiedje, 2007; Ge *et al*., 2008). Here, we employed a more powerful phylogenetic approach that takes into account different levels of similarity between different pairs of taxa (Lozupone and Knight, 2005) to compare more subtle differences among the samples. Consequently, a significant correlation was observed between bacterial community variation and geographic distance (Fig. 6). Meanwhile, there was little taxonomic overlap across the sites (Table S5). These results suggest that geographic distance is an indicator of bacterial dispersal among sediments. The geographic distance explained more variation in community structure than any other individual factor (Fig. 7). The unexplained variation may be due to additional factors not measured in this study, such as sediment temperature and redox state (Pett-Ridge and Firestone, 2005), which can influence bacterial communities.

In contrast, a recent continent-scale survey of soils showed that geographic distance was not correlated with changes in community structure (Chu *et al*., 2010), whereas Ge and colleagues observed 60.3% of the variation among communities was explained by spatial isolation (Ge *et al*., 2008). The former study was performed in an open environment, where wind, animal and other vectors may spread the bacteria, leading to random spatial distributions (Green and Bohannan, 2006); another similar study also showed that when the geographic distance increased, the correlation between community dissimilarity and geographic distance disappeared (Griffiths *et al*., 2011). Similarly, no ammonia-oxidizing bacterial diversification was detected in salt marsh sediments across the continental scale (Martiny *et al*., 2011). However, the latter study only collected samples from three locations (Ge *et al*., 2008), limiting its statistical power, especially because the relative influence of historical and environmental factors depends on the sampling scale (Ricklefs, 2004; Martiny *et al*., 2006). A small-scale (0.03 m to 300 m) survey in salt marsh sediments indicated that, at least on this scale and in this system, the bacterial taxa–area relationship was primarily driven by environmental heterogeneity (Horner-Devine *et al*., 2004); similar results were obtained in a gradient of hypersaline lake sediments along a 140 m transect (Hollister *et al*., 2010). Therefore, studies need to be performed across a range of spatial scales and in a range of systems, as is being performed in projects such as the Earth Microbiome Project (Gilbert *et al*., 2010).

In conclusion, this study represents a large attempt to investigate bacterial spatial patterns in pristine sediments across the Tibetan Plateau. We show that bacterial community structure, phylotype richness and phylogenetic diversity are predicted by sediment pH, even within a narrow pH range. This is the first study to demonstrate that pH predicts microbial community structure in alkaline sediments, mirroring results in acidic soils. Our results clearly show that local geochemical features are the dominant factors in driving the bacterial community variation, whereas geographic distances as a single factor also a larger contributor in community variation across a regional spatial scale (about 1670 km). From a practical point, future research in this area would benefit from using appropriate sampling scale and relative less disturbed ecosystem to study microbial biogeography (Green *et al*., 2004; Ramette and Tiedje, 2007). These observations thus provide baseline information for predicting regional-scale responses to future environmental changes.

## Experimental procedure

### Sample collection

The Tibetan Plateau lies at a critical and sensitive junction of three climatic systems: the East Asian Monsoon, the cold polar airflow from the Siberian high pressure, and the Indian monsoon (Jiang *et al*., 2007). The ages of lakes located at the Tibetan Plateau were estimated to be 2 to 8 million years (Zheng and Yao, 2004), sediments in lake record past climatic and environmental changes.

In the summer of 2010, we collected 17 surface sediments (0–5 cm) from 15 lakes across the Tibetan Plateau, and duplicate samples were selected from two lakes to compare local variances of bacterial community. The lakes were chosen to cover a salinity gradient from 0.32 g l^−1^ (0.03%, freshwater) to 308.0 g l^−1^ (30.8%, hypersaline). The sediment samples were mixed thoroughly and immediately shipped to lab on ice packs. Subsamples were archived at 4°C for sediment geochemical characterization and −80°C for genomic DNA extraction. GPS coordinates recorded at each sampling point (varied from 29°13′–34°36′N, 79°47′–96°47′E), imported into the NOAA website (http://www.nhc.noaa.gov/gccalc.shtml) to calculate the pairwise geographic distance. The pH values were measured using pH strips with 1:1 (wt/vol) of sediment to water. Sediments were immediately freeze-dried to detect geochemical characteristics. Concentrations of sodium (Na^+^) and magnesium (Mg^2+^) ions and TP were measured using inductively coupled plasma mass spectrometry (ICP-MS) (ELAN 9000/DRC-e, PerkinElmer, USA). TC and total nitrogen (TN) were measured by CN Analyzer (Vario Max CN, Elementar, Germany).

### DNA extraction and purification

Community DNA was extracted from sediments using a FastDNA® Spin kit (Bio 101, Carlsbad, CA, USA) according to the manufacturer's protocol. The raw DNA was purified using a 0.8% (wt/vol) low melting point agarose gel. The DNA bands were excised, then extracted using an agarose gel DNA purification kit (TaKaRa), and quantified with a NanoDrop ND-1000 spectrophotometer (NanoDrop Technologies, Wilmington, DE, USA). Purified DNA was stored at −20°C until use.

### Bacterial 16S rRNA amplification and 454 sequencing

An aliquot (50 ng) of purified DNA from each sample were used as template for amplification; the V4–V5 hypervariable regions of bacterial 16S rRNAs (*Escherichia coli* positions 515–907, Biddle *et al*., 2008) were amplified using the primer set: F515: GTGCCAGCMGCCGCGG with the Roche 454 ‘A’ pyrosequencing adapter and a unique 7 bp barcode sequence, and primer R907: CCGTCAATTCMTTTRAGTTT with the Roche 454 ‘B’ sequencing adapter at the 5′-end of each primer respectively. Each sample was amplified in triplicate with 50 µl reaction under the following conditions: 30 cycles of denaturation at 94°C for 30 s, annealing at 55°C for 30 s, and extension at 72°C for 30 s; with a final extension at 72°C for 10 min. PCR products were pooled together and purified by Agarose Gel DNA purification kit (TaKaRa) as described above.

The PCR products from each sample were combined in equimolar ratio in a single tube and to run on a Roche FLX 454 pyrosequencing machine (Roche Diagnostics Corporation, Branford, CT, USA), producing reads from the forward direction F515.

### Processing of pyrosequencing data

Data were processed as described previously (Fierer *et al*., 2008; He *et al*., 2010), using the Quantitative Insights Into Microbial Ecology (QIIME) pipeline (http://qiime.sourceforge.net/) (Caporaso *et al*., 2010). Specifically, bacterial sequences with the same barcode were assigned into the same sample, and then the barcode and primer sequences were removed. Only the first 350 bp after primer-F515 of each sequence were included to improve reads quality for further analysis. Bacterial phylotypes were identified using uclust (Edgar, 2010) and assigned to operational taxonomic units (OTUs, 97% similarity). Representative sequences from each phylotype were aligned using PyNAST (DeSantis *et al*., 2006a), and the most abundant sequence in the OTU was selected as the representative sequence. Taxonomic identity of each phylotype was determined using the Greengenes database (DeSantis *et al*., 2006b). To correct for sampling effort, we used a randomly selected subset of 4000 sequences per sample to calculate distances between samples. The differences in overall community composition between each pair of samples were determined using the UniFrac metric (Lozupone and Knight, 2005), which provides a more robust index of community distance than taxon-based methods.

### Statistical analysis

Phylogenetic diversities were estimated using Faith's index, which incorporates the phylogenetic breadth across taxonomic levels (Faith, 1992). The relationships between the taxonomic diversity for the group with geochemical features were tested with linear regression analyses using spss 17.0 for Windows. Raw environmental data were standardized to make the different environmental factors comparable. Euclidean distances were used to construct a similarity matrix for sediment properties. Partial Mantel tests were used to calculate the correlation between the UniFrac distances of bacterial communities and the sediment characteristics or geographic distance in PASSaGE (Rosenberg and Anderson, 2011). BioEnv and canonical correspondence analysis (CCA) were also used to identify the abiotic factors most important to bacterial community composition, and they were used to construct the sediment property matrix for variation partitioning analysis in R v.2.8.1 with the vegan package (R Development Core Team, 2008).

## Supporting information

**Fig. S1.** Lake water salinity in relative to bacterial phylotype richness (a) and phylogenetic diversity (b) based on 97% sequence similarity in Tibetan Plateau lake sediments. The communities were randomly sampled at the 4000 sequences level.

**Fig. S2.** Sediment pH in relative to*Archaea* phylotype richness based on 97% sequence similarity in Tibetan Plateau lake sediments. The communities were randomly sampled at the 4000 sequences level.

**Table S1.** Sediment physical and chemical properties and lake water salinity.

**Table S2.** Pairwise geographic distance (km) matrix.

**Table S3.** Relative average abundances of phyla across all sediments and sediments grouped into various pH categories (values represent % of total non-redundant sequences). Asterisks indicate sequences classified to the domain Bacteria, but not to a specific phylum.

**Table S4.** Correlations (*r*) betweenbacterial phylotype richness and phylogenetic diversity andsediment and site characteristics and lake water salinity. Valuesin bold indicate significant correlations (*P* < 0.05).

**Table S5** The detected OUTs, percentages of overlap, unique and diversity indices for each sample per 4000 sequences.
