# Supplementary material for: Geographic distance and pH drive bacterial distribution in alkaline lake sediments across Tibetan Plateau
Source: Environ Microbiol. 2012 Sep;14(9):2457–66. doi: 10.1111/j.1462-2920.2012.02799.x (PMC3477592; doi:10.1111/j.1462-2920.2012.02799.x)

**Supplemental data summary**

**Table S1** Sediment physical and chemical properties and lake water salinity

| Lakes | ID | Location | Altitude (m) | Lake water salinity | pH | TC % | TN % | C/N | P (g/kg) | Na | Mg |
| --- | --- | --- | --- | --- | --- | --- | --- | --- | --- | --- | --- |
|  |  |  |  | g/L |  |  |  |  |  | mg/kg | |
| Awong Co | AWC | N 32°46′ E 81°44′ | 4374 | 87.1 | 9.49 | 7.88 | 0.140 | 56.3 | 0.24 | 13.9 | 84.6 |
| Aiyong Co | AYC | N 33°37′ E 80°34′ | 4292 | 71.3 | 9.73 | 4.73 | 0.134 | 35.3 | 0.45 | 13.7 | 38.0 |
| BanGong Co | BGC | N 33°29′ E 79°48′ | 4167 | 2.7 | 8.16 | 8.12 | 0.512 | 15.9 | 0.47 | 6.2 | 12.1 |
| Bieruoze Co | BRZC | N 32°26′ E 82°57′ | 4324 | 115.1 | 9.27 | 7.32 | 0.113 | 64.8 | 0.32 | 11.9 | 58.5 |
| Darebu Co | DRBC | N 32°28′ E 83°13′ | 4436 | 109.8 | 9.03 | 11.80 | 0.487 | 24.2 | 0.41 | 4.4 | 34.5 |
| Daze Co | DZC | N 31°51′ E 87°46′ | 4393 | 40.6 | 10.37 | 4.45 | 0.189 | 23.5 | 0.43 | 13.9 | 24.8 |
| Gongzhu Co | GZC | N 30°35′ E 82°08′ | 4710 | 5.4 | 9.65 | 13.20 | 0.933 | 14.1 | 0.56 | 11.1 | 36.2 |
| Kunzhong Co | KZC1 | N 33°07′ E 80°23′ | 4266 | 64.3 | 9.12 | 5.72 | 0.424 | 13.5 | 0.56 | 11.6 | 19.6 |
| Kunzhong Co | KZC2 | N 33°02′ E 80°23′ | 4266 | 64.3 | 8.49 | 9.29 | 0.589 | 15.8 | 0.65 | 10.4 | 15.2 |
| Lubu Co | LBC1 | N 33°06′ E 80°10′ | 4271 | 45.8 | 6.88 | 9.85 | 0.590 | 16.7 | 0.52 | 10.0 | 9.6 |
| Lubu Co | LBC2 | N 33°07′ E 80°15′ | 4271 | 45.8 | 8.00 | 9.03 | 0.517 | 17.5 | 0.49 | 13.3 | 10.1 |
| Lang Co | LC | N 29°13′ E 87°24′ | 4213 | 1.97 | 9.60 | 1.69 | 0.141 | 12.0 | 0.79 | 11.9 | 18.8 |
| Longmu Co | LMC | N 34°35′ E 80°22′ | 4933 | 173.6 | 8.40 | 4.37 | 0.023 | 190.0 | 0.41 | 11.9 | 26.8 |
| Rebang Co | RBC | N 33°02′ E 80°29′ | 4250 | 70.0 | 9.70 | 3.68 | 0.154 | 23.9 | 0.43 | 17.1 | 28.5 |
| Ranwu Co | RWC | N 29°27′ E 96°47′ | 3850 | 0.32 | 8.24 | 1.15 | 0.063 | 18.3 | 0.53 | 14.1 | 17.7 |
| Sumxi Co | SMXC | N 34°36′ E 80°15′ | 4975 | 0.43 | 8.04 | 3.48 | 0.091 | 38.2 | 0.48 | 9.6 | 15.2 |
| Zhacang Chaka | ZCCK | N 32°32′ E 82°26′ | 4400 | 308.0 | 9.50 | 5.32 | 0.223 | 23.9 | 0.46 | 17.3 | 29.2 |

**Table S2** Pairwise geographic distance (km) matrix

|  | AWC | AYC | BGC | BRZC | DRBC | DZC | GZC | KZC1 | KZC2 | LBC1 | LBC2 | LC | LMC | RBC | RWC | SMXC | ZCCK |
| --- | --- | --- | --- | --- | --- | --- | --- | --- | --- | --- | --- | --- | --- | --- | --- | --- | --- |
| AWC |  |  |  |  |  |  |  |  |  |  |  |  |  |  |  |  |  |
| AYC | 128 |  |  |  |  |  |  |  |  |  |  |  |  |  |  |  |  |
| BGC | 197 | 73 |  |  |  |  |  |  |  |  |  |  |  |  |  |  |  |
| BRZC | 120 | 245 | 317 |  |  |  |  |  |  |  |  |  |  |  |  |  |  |
| DRBC | 143 | 267 | 339 | 26 |  |  |  |  |  |  |  |  |  |  |  |  |  |
| DZC | 548 | 667 | 741 | 430 | 406 |  |  |  |  |  |  |  |  |  |  |  |  |
| GZC | 238 | 336 | 384 | 212 | 227 | 524 |  |  |  |  |  |  |  |  |  |  |  |
| KZC1 | 132 | 33 | 68 | 252 | 275 | 679 | 321 |  |  |  |  |  |  |  |  |  |  |
| KZC2 | 130 | 39 | 72 | 250 | 273 | 678 | 315 | 4 |  |  |  |  |  |  |  |  |  |
| LBC1 | 151 | 48 | 54 | 271 | 295 | 699 | 331 | 21 | 21 |  |  |  |  |  |  |  |  |
| LBC2 | 144 | 41 | 59 | 264 | 287 | 691 | 327 | 12 | 14 | 5 |  |  |  |  |  |  |  |
| LC | 669 | 796 | 864 | 555 | 539 | 293 | 531 | 796 | 793 | 813 | 806 |  |  |  |  |  |  |
| LMC | 239 | 137 | 134 | 339 | 354 | 727 | 468 | 166 | 170 | 166 | 164 | 892 |  |  |  |  |  |
| RBC | 121 | 38 | 81 | 291 | 264 | 669 | 308 | 13 | 10 | 31 | 23 | 784 | 173 |  |  |  |  |
| RWC | 1478 | 1597 | 1670 | 1359 | 1335 | 930 | 1414 | 1609 | 1608 | 1629 | 1621 | 910 | 1647 | 1598 |  |  |  |
| SMXC | 246 | 140 | 131 | 347 | 363 | 737 | 473 | 165 | 172 | 166 | 164 | 902 | 11 | 175 | 1657 |  |  |
| ZCCK | 70 | 196 | 267 | 50 | 74 | 480 | 212 | 202 | 200 | 221 | 214 | 761 | 228 | 190 | 1409 | 305 |  |

**Table S3** Relative average abundances of phyla across all sediments and sediments grouped into various pH categories (values represent % of total non-redundant sequences). Asterisks indicate sequences classified to the domain Bacteria, but not to a specific phylum.

| Phylum | All | pH 6.88 | pH 8.0-8.5 | pH 9.0-9.5 | pH 9.5-10.0 | pH 10.37 |
| --- | --- | --- | --- | --- | --- | --- |
| *Bacteroidetes* | 14.248 | 10.914 | 9.621 | 22.445 | 12.971 | 9.466 |
| *Firmicutes* | 13.236 | 4.598 | 8.357 | 13.637 | 20.634 | 19.554 |
| *Gammaproteobacteria* | 9.505 | 7.200 | 9.711 | 10.072 | 9.320 | 8.483 |
| *Deltaproteobacteria* | 8.054 | 4.731 | 6.253 | 10.095 | 8.215 | 11.335 |
| *Betaproteobacteria* | 6.596 | 9.682 | 13.717 | 3.058 | 1.178 | 0.144 |
| *Actinobacteria* | 6.449 | 11.419 | 11.261 | 2.462 | 4.112 | 1.893 |
| *Alphaproteobacteria* | 6.313 | 8.508 | 8.680 | 4.486 | 5.023 | 4.218 |
| *Chloroflexi* | 3.069 | 1.604 | 2.406 | 3.359 | 3.806 | 4.122 |
| *Cyanobacteria* | 3.018 | 5.116 | 3.775 | 3.877 | 0.979 | 0.240 |
| *Deinococcus-Thermus* | 2.938 | 0.032 | 0.342 | 2.31 | 4.41 | 18.64 |
| *Acidobacteria* | 1.039 | 5.343 | 1.373 | 0.330 | 0.406 | 0.815 |
| *Bacteria** | 20.23 | 22.71 | 17.50 | 19.50 | 25.07 | 18.52 |
| *Planctomycetes* | 1.024 | 2.002 | 1.725 | 0.289 | 0.794 | 0.431 |
| *Nitrospira* | 0.375 | 1.137 | 0.837 | 0.013 | 0.037 | < 0.001 |
| *Spirochaetes* | 0.184 | < 0.001 | 0.021 | 0.458 | 0.178 | < 0.001 |
| *WS3* | 0.154 | 0.139 | 0.227 | 0.087 | 0.153 | 0.072 |
| *Gemmatimonadetes* | 0.114 | 0.657 | 0.174 | 0.039 | 0.007 | < 0.001 |
| *Crenarchaeota* | 0.102 | 1.270 | 0.024 | 0.027 | 0.023 | 0.096 |
| *Verrucomicrobia* | 0.068 | 0.126 | 0.066 | 0.092 | 0.019 | 0.096 |
| *Euryarchaeota* | 0.055 | 0.025 | 0.013 | 0.049 | 0.145 | < 0.001 |
| *OP10* | 0.040 | 0.063 | 0.064 | 0.026 | 0.019 | 0.024 |
| *Tenericutes* | 0.017 | < 0.001 | 0.003 | 0.010 | 0.046 | 0.048 |
| *TM7* | 0.010 | 0.006 | 0.016 | 0.008 | 0.006 | < 0.001 |
| *BRC1* | 0.009 | 0.013 | 0.025 | < 0.001 | < 0.001 | < 0.001 |
| *Deferribacteres* | 0.009 | < 0.001 | 0.024 | < 0.001 | < 0.001 | < 0.001 |
| *OP11* | 0.008 | 0.006 | 0.014 | 0.008 | < 0.001 | < 0.001 |
| *OD1* | 0.002 | < 0.001 | 0.004 | 0.002 | < 0.001 | < 0.001 |

**Table S4** Correlations (*r*) between bacterial phylotype richness and phylogenetic diversity and sediment and site characteristics and lake water salinity. Values in bold indicate significant correlations (*p* < 0.05).

| *r* | Altitude | Latitude | Lake water salinity | pH | TC | TN | C/N | P | Na^+^ | Mg^2+^ |
| --- | --- | --- | --- | --- | --- | --- | --- | --- | --- | --- |
| Richness | -0.178 | 0.261 | -0.303 | **-0.706** | -0.124 | -0.037 | -0.177 | 0.150 | 0.020 | -0.402 |
| Diversity | -0.200 | 0.261 | -0.315 | **-0.731** | -0.069 | 0.033 | -0.234 | 0.173 | -0.074 | -0.461 |

**Table S5** The detected OUTs, percentages of overlap, unique and diversity indices for each sample per 4,000 sequences.

| Sample | AWC | AYC | BGC | BRZC | DRBC | DZC | GZC | KZC1 | KZC2 | LBC1 | LBC2 | LC | LMC | RBC | RWC | SMXC | ZCCK |
| --- | --- | --- | --- | --- | --- | --- | --- | --- | --- | --- | --- | --- | --- | --- | --- | --- | --- |
| AWC | **56.68*** | *6.73* | *0.38* | *12.27* | *1.94* | *8.09* | *4.57* | *3.20* | *1.42* | *0.19* | *0.46* | *1.57* | *0.65* | *12.79* | *0.14* | *0.18* | *1.35* |
| AYC |  | **67.68** | *0.26* | *7.24* | *1.15* | *5.86* | *2.31* | *2.82* | *1.67* | *0.60* | *0.85* | *1.47* | *1.50* | *7.89* | *0.43* | *0.29* | *2.02* |
| BGC |  |  | **69.61** | *0.42* | *4.26* | *0.45* | *1.90* | *3.74* | *3.21* | *3.80* | *5.07* | *3.79* | *0.42* | *0.71* | *1.71* | *2.66* | *1.97* |
| BRZC |  |  |  | **56.15** | *1.83* | *7.48* | *4.52* | *3.78* | *1.92* | *0.22* | *0.72* | *1.46* | *1.05* | *11.14* | *0.24* | *0.41* | *1.41* |
| DRBC |  |  |  |  | **67.33** | *1.94* | *5.76* | *6.50* | *4.67* | *2.90* | *4.23* | *3.79* | *0.48* | *1.83* | *0.73* | *1.24* | *2.23* |
| DZC |  |  |  |  |  | **53.41** | *6.51* | *5.23* | *3.16* | *0.80* | *1.14* | *2.74* | *0.87* | *11.43* | *0.61* | *0.44* | *1.99* |
| GZC |  |  |  |  |  |  | **58.39** | *8.37* | *4.27* | *0.76* | *1.77* | *4.09* | *0.23* | *6.15* | *0.37* | *0.31* | *1.16* |
| KZC1 |  |  |  |  |  |  |  | **49.00** | *11.50* | *2.77* | *5.66* | *6.16* | *1.49* | *4.31* | *1.69* | *1.56* | *5.12* |
| KZC2 |  |  |  |  |  |  |  |  | **52.14** | *3.08* | *6.84* | *5.10* | *1.06* | *2.84* | *2.75* | *1.66* | *7.14* |
| LBC1 |  |  |  |  |  |  |  |  |  | **74.85** | *5.37* | *2.33* | *1.48* | *0.80* | *4.41* | *3.34* | *2.72* |
| LBC2 |  |  |  |  |  |  |  |  |  |  | **66.86** | *3.81* | *1.71* | *1.26* | *3.63* | *2.67* | *4.54* |
| LC |  |  |  |  |  |  |  |  |  |  |  | **65.15** | *1.03* | *1.75* | *2.12* | *2.96* | *2.42* |
| LMC |  |  |  |  |  |  |  |  |  |  |  |  | **84.31** | *0.83* | *1.21* | *0.83* | *3.13* |
| RBC |  |  |  |  |  |  |  |  |  |  |  |  |  | **45.88** | *0.61* | *0.47* | *1.76* |
| RWC |  |  |  |  |  |  |  |  |  |  |  |  |  |  | **74.95** | *7.02* | *2.42* |
| SMXC | |  |  |  |  |  |  |  |  |  |  |  |  |  |  | **76.87** | *1.87* |
| ZCCK |  |  |  |  |  |  |  |  |  |  |  |  |  |  |  |  | **69.20** |
| OTUs | 1392 | 1445 | 1257 | 1398 | 1555 | 1174 | 1216 | 1543 | 1473 | 2239 | 2118 | 1455 | 1389 | 1166 | 2303 | 2028 | 1844 |
| PD^&^ | 102.3 | 103.4 | 101.1 | 98.6 | 131.2 | 91.8 | 90.0 | 120.4 | 113.0 | 180.7 | 165.3 | 110.3 | 101.7 | 88.6 | 180.9 | 157.4 | 140.1 |

^*^Values in boldface type represent unique genes in each sample, and italic type represent overlapped genes between two samples;

^&^ Phylogenetic diversity

**Fig. S1** Lake water salinity in relative to bacterial phylotype richness (a) and phylogenetic diversity (b) based on 97% sequence similarity in Tibetan Plateau lake sediments. The communities were randomly sampled at the 4,000 sequences level.


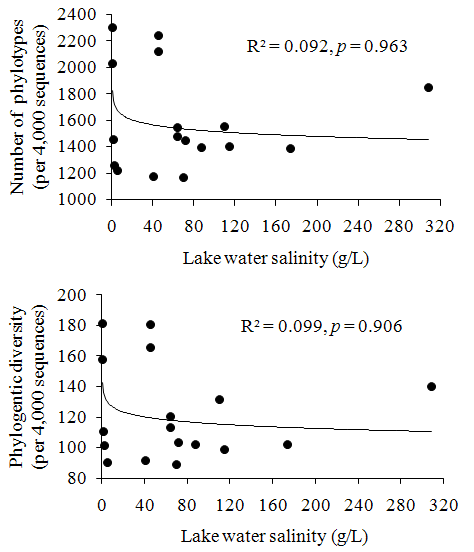


**Fig. S2** Sediment pH in relative to *Archaea* phylotype richness based on 97% sequence similarity in Tibetan Plateau lake sediments. The communities were randomly sampled at the 4,000 sequences level.


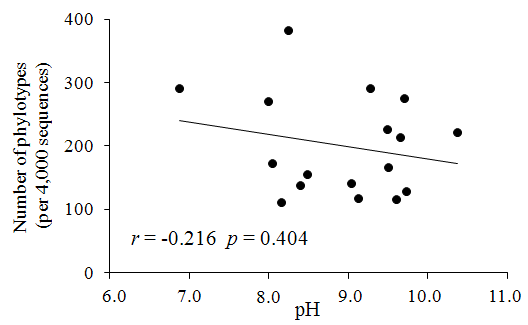

Supplement: Supplementary file 1 [file emi0014-2457-SD1.doc]
